# Effectiveness of Biosecurity Measures in Preventing Badger Visits to Farm Buildings

**DOI:** 10.1371/journal.pone.0028941

**Published:** 2011-12-29

**Authors:** Johanna Judge, Robbie A. McDonald, Neil Walker, Richard J. Delahay

**Affiliations:** 1 The Food & Environment Research Agency, Sand Hutton, York, Yorkshire, United Kingdom; 2 Environment and Sustainability Institute, University of Exeter, Cornwall Campus, Penryn, Cornwall, United Kingdom; University of Georgia, United States of America

## Abstract

**Background:**

Bovine tuberculosis caused by *Mycobacterium bovis* is a serious and economically important disease of cattle. Badgers have been implicated in the transmission and maintenance of the disease in the UK since the 1970s. Recent studies have provided substantial evidence of widespread and frequent visits by badgers to farm buildings during which there is the potential for close direct contact with cattle and contamination of cattle feed.

**Methodology:**

Here we evaluated the effectiveness of simple exclusion measures in improving farm biosecurity and preventing badger visits to farm buildings. In the first phase of the study, 32 farms were surveyed using motion-triggered infrared cameras on potential entrances to farm buildings to determine the background level of badger visits experienced by each farm. In the second phase, they were divided into four treatment groups; “Control”, “Feed Storage”, “Cattle Housing” and “Both”, whereby no exclusion measures were installed, exclusion measures were installed on feed storage areas only, cattle housing only or both feed storage and cattle housing, respectively. Badger exclusion measures included sheet metal gates, adjustable metal panels for gates, sheet metal fencing, feed bins and electric fencing. Cameras were deployed for at least 365 nights in each phase on each farm.

**Results:**

Badger visits to farm buildings occurred on 19 of the 32 farms in phase one. In phase two, the simple exclusion measures were 100% effective in preventing badger entry into farm buildings, as long as they were appropriately deployed. Furthermore, the installation of exclusion measures also reduced the level of badger visits to the rest of the farmyard. The findings of the present study clearly demonstrate how relatively simple practical measures can substantially reduce the likelihood of badger visits to buildings and reduce some of the potential for contact and disease transmission between badgers and cattle.

## Introduction

Agricultural buildings may be attractive to wildlife for a variety of reasons. They can provide shelter, particularly during the winter to escape harsh temperatures [1]. Foraging opportunities arise from the availability of stored livestock feed and harvested crops, particularly for rodents which in turn may attract predators [2], [3]. In addition to the potential for costly losses of stored feed and crops, wildlife activity may also increase the risk of spreading pathogens of agricultural and zoonotic importance such as *Brucella*, *Trichinella* [4], *Mycobacterium avium paratuberculosis* [5] and *Cryptosporidium* [6]. Disease risks may arise as a result of direct contact between wildlife and livestock or contamination by wildlife of buildings, equipment and feed. For example, it has been estimated that individual cattle or sheep could come into contact with 1626 and 814 rodent or bird droppings respectively in stored feed over one winter [7]. Developing simple methods of excluding wildlife from farm buildings may therefore be a useful tool in the mitigation of disease transmission risk between livestock and wild hosts.

The Eurasian badger (*Meles meles*) is the principal wildlife reservoir of *Mycobacterium bovis* (the causative agent of bovine tuberculosis infection) in the UK and Ireland [8], [9]. The failure to eradicate bovine tuberculosis (TB) from cattle in these countries is hampered by the transmission of infection between badgers and cattle. Infectious badgers can excrete *M. bovis* bacilli in faeces, urine, sputum and exudate from wounds and abscesses [10]. Contact with badgers or their excretions may therefore present opportunities for the infection of cattle [11], [12].

The principal route by which infection is transmitted from badgers to cattle is not clear. From the few studies that have been conducted, direct contact between badgers and grazing cattle appears relatively infrequent [13], [14]. In contrast, several studies have demonstrated contamination of pasture with badger faeces and urine [12], [13], [15]–[17], and subsequent calculations suggest potentially significant risks of exposure to cattle [18]. More recent research suggests that the potential for disease transmission to cattle as a result of badger activity in farm buildings may also be substantial. Several studies have now demonstrated that badger visits to farm buildings are frequent and widespread in the southwest of England [19]-[23]. During these visits badgers have been observed foraging on stored feed, invertebrates and vertebrate prey, collecting bedding, and coming to within 2m of housed cattle [19],[21],[24]. Observations of badgers defecating, urinating and grooming in buildings, sometimes in direct contact with cattle feed, provide evidence of the potential for indirect transmission of *M. bovis* via contamination of this environment [19], [21], [24].

Numerous studies have been conducted to investigate methods of reducing contact between wildlife and livestock on pasture, with varying degrees of success. For example fitting electric shock collars to wolves, which were activated when the wolves came within a certain distance of the protected area [25] and using acoustic frightening devices to deter coyotes [26] in order to reduce predation on sheep, ultrasonic devices and water jets to deter badgers [27], lasers to disperse deer [28], [29] and electric fencing to keep deer [30] and badgers [31] out of crop fields. However, to date, little research has been aimed specifically at keeping wildlife out of farm buildings, although a notable exception was the localised evaluation of the use of electric fencing to reduce badger visits [32].

Here we describe the results of an experimental study to investigate the effectiveness of a range of simple exclusion measures on the level and frequency of badger visits to farm yards and buildings. The aims were to determine (i) if simple exclusion measures deter badger visits to farmyards and buildings and (ii) if exclusion measures cause displacement of badger activity to unprotected buildings.

## Methods

### Study farm selection

The study was undertaken in Gloucestershire, a county of southwest England with a high incidence of bovine TB in cattle. Potential study farms that had not been the subject of badger culling during the Randomised Badger Culling Trial (RBCT) from 1998 to 2005 inclusive (Bourne et al. 2007), and which were under annual TB testing of their cattle herds, were randomly selected from VETNET (The UK Department for the Environment, Food and Rural Affairs (Defra) bovine TB control and surveillance database). From this sample, we selected 32 farms with a herd size of at least 30 animals, which were kept indoors for at least part of the year, and where concentrates or cereal feed (e.g. cake, grain, barley, sugar beet) were stored on site but separately from housed cattle.

### Experimental design

The experiment consisted of two phases, both lasting at least 365 days on each farm. During an initial surveillance phase (between 1st February 2007 and 31st August 2008) we established the background frequency of badger visits to all farms. During the second phase (between 1st February 2008 and 31st August 2009) we investigated the effect on badger visits of installing exclusion measures on farm buildings. For logistical reasons surveillance was initiated on different dates on individual farms, and consequently the periods of surveillance on each farm were not simultaneous.

Clearly we could only measure the effects of exclusion measures on farms where badgers were found to visit. Hence, while all 32 farms were monitored in both the first and second phases of the experiment, only those which experienced badger visits during the first background surveillance phase are included in the statistical analyses described below.

### Surveillance

Infra-red, motion-triggered, digital still cameras (Leaf River IR3-BU, Vibrashine Inc., Taylorsville MS, USA; Stealth Cam 1430IR, Stealth Cam LLC, Grand Prairie TX, USA and Game Spy I40, Moultrie Feeders, Alabaster AL, USA) were deployed at potential badger access points to cattle sheds, feed stores, and silage clamps on all study farms. The positioning of cameras was constrained by the need to avoid them being damaged by livestock or machinery during normal farm working practices. Between four and thirteen cameras were deployed on each farm, depending on the size and the number of buildings and potential entrance points for badgers. The cameras were operational nightly throughout both phases of the experiment.

Memory cards, with at least 1Gb of storage capacity and batteries were replaced every two weeks. Images were downloaded from retrieved memory cards and all observations of badgers and other wildlife were catalogued using Extensis Portfolio 8 software (Extensis, Portland OR, USA). The date, time, farm ID, individual camera identity, type of building (feed store, silage clamp or cattle housing), and species observed was recorded for each observation. During phase 2, if an image clearly showed the exclusion measure was not in use, or otherwise allowed badger access (e.g. was damaged), on particular nights, this was also recorded. Images documenting badger visits were also allocated to one of two categories. Where a badger was clearly evident either entering or already inside a building, the observation was classified as a ‘building visit’, but where it was neither inside nor entering a building this was deemed a ‘farmyard visit’.

### Badger exclusion measures

In order to investigate the effects of installing badger exclusion measures on farm buildings, the study employed a factorial design (Table 1). Each farm was allocated to one of four experimental treatments where farms had: no exclusion measures, measures to reduce visits to cattle housing and associated feed troughs only, measures to reduce visits to feed stores (including silage clamps) only or measures to reduce visits to cattle housing (including feed troughs), and feed stores (including silage clamps). These treatments were each replicated eight times (n = 32 farms). Treatment was allocated to each farm towards the end of the initial surveillance phase, using a randomised complete block design to ensure an even distribution of farms with respect to the frequency of badger visits in phase 1 across the four treatment groups.

**Table 1:** The factorial design of the study, showing the exclusion measure combinations by treatment.

	Treatment			
	Control	Cattle Housing	Feed Stores	Both
Measures on:				
Cattle Housing	No	Yes	No	Yes
Feed Stores	No	No	Yes	Yes

**Figure 1:**
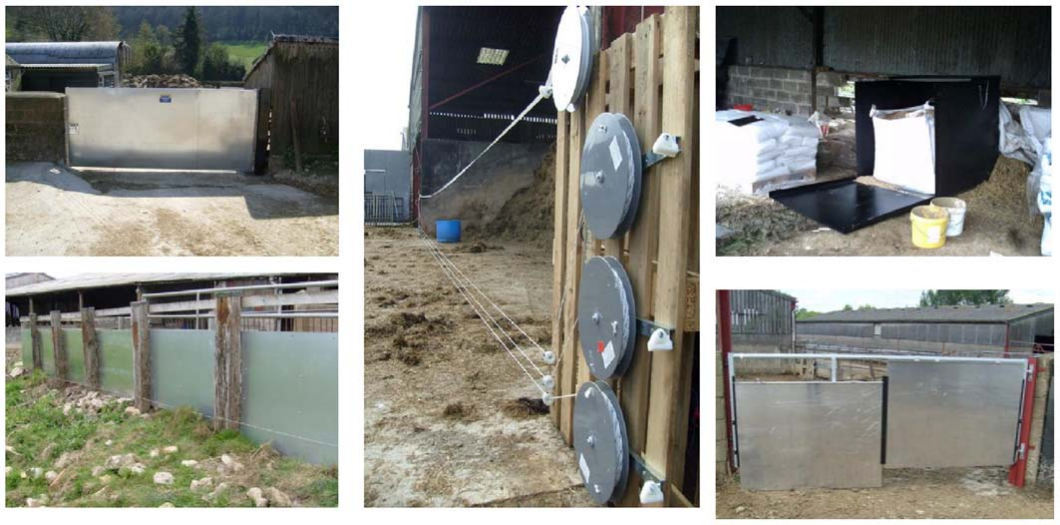
Examples of badger exclusion measures: solid aluminium sheeted gate (top left), aluminium sheeting installed on rail fence (bottom left), retractable electric fencing (middle), front and top opening aluminium feed bin (top right) and rail gate with adjustable galvanised aluminium panels (bottom right).

The badger exclusion measures were individually tailored to fit the requirements of each farm and sought to secure every potential entrance point on each selected facility. The five main exclusion measures used were galvanised aluminium sheeted metal gates, adjustable galvanised aluminium sheeted panels (which could be moved up or down) on gates, galvanised aluminium sheeted fencing, aluminium feed bins and electric fencing (Figure 1). A full list of measures employed on each farm is given in Table S1. Other measures installed on some farms included sheeted gates with hinged flaps, roller doors, metal sheets attached to angled feed troughs and sheeted wheeled barriers. Gates and fences were constructed and fitted so that the gap between the bottom and the ground was less than approximately 7.5cm as this was considered to be sufficiently low to prevent badger access. Gates with two or three adjustable solid panels that could be raised or lowered were employed on uneven ground and deep litter.

Electric fencing (either fixed or retractable) was installed on farms where permanent gates or panels were not suitable, such as on very uneven ground or in areas where farm machinery access would have been compromised. The area beneath fixed-position electric fences over rough ground was sprayed with herbicide to retard vegetation growth which could otherwise cause the fence to short-circuit. Retractable electric fences were installed on silage clamps and across farmyards that were too wide for conventional gates and required frequent farm machinery access. The electric fence strands were held on self-tensioning reel systems, fixed to an insulated rod, which could be pulled across gaps of up to 20 metres. The height of the bottom three strands of fencing were 10, 15 and 20 cm above the ground as specified in designs that have been demonstrated to effectively exclude badgers [31], [32]. A fourth non-electrified strand was placed at a height of approximately 122cm to increase the visibility of the fence as a safety measure to prevent farm workers accidentally driving through, or tripping over, the lower strands.

During the fortnightly building surveys, any observed damage to badger exclusion measures was recorded. In addition, details of whether the measures were maintained *in situ* by farmers were also recorded from the images taken during camera trapping where possible. Although this study was not designed to quantify the extent to which exclusion measures were employed and maintained by farmers, we attempted to gain some insights by calculating the number of nights that any measure was observed (from digital images) to be in use as a percentage of the total number of nights when the camera was activated. A conservative approach was employed, whereby all digital images from nights when multiple images suggested that measures were only adequately employed for part of the night were excluded. In addition, as we would expect more wildlife visits to take place (and therefore to be recorded in digital images) when exclusion measures were not adequately employed, we also excluded all images which contained wildlife. Hence, all remaining images were likely to have been triggered by non-wildlife events (e.g. wind-blown leaves) which are likely to have taken place independently of whether exclusion measures were correctly employed. This approach yielded a minimum estimate of the number of nights when exclusion measures were not adequately employed because we were unable to determine if the measures had been in use on those nights when cameras were not triggered.

### Statistical Analyses

#### Camera level analyses

In order to assess the effect of fitting exclusion measures on buildings, images from each camera were examined for evidence of badger visits. Each observation in this analysis represented whether or not a badger visit was observed by a given camera on a given night (a camera-night). If a camera was known not to have been working on specific nights, those nights for that camera were omitted from the analyses.

Variations in the binary variable “building visit” (1 = 1 or more visits observed on a given camera night and 0  =  no visits observed on a given camera night) were related to potential explanatory variables using a Generalised Linear Mixed Model (GLMM; GenStat for Windows, Version 13, VSN International, Hemel Hempstead, UK). Factors affecting the probability of a building visit were modelled with a binomial distribution using a logit-link transformation [33]. Fixed effect explanatory variables were season (spring  =  March to May, summer  =  June to August, autumn  =  September to November and winter  =  December to February inclusive), experimental phase (1  =  pre-treatment phase, 2  =  treatment phase) and building type (cattle housing or feed store). The model included all observations from phase 1 and phase 2 in order to allow for within-farm and year-to-year variation to be accounted for. A further explanatory variable was treatment status, which described whether any exclusion measures were in place on the entire farm (i.e. either no exclusion measures were present, measures were in place on the building covered by that camera, or they were in place somewhere else on the farm). For the purposes of these analyses, all exclusion measures were considered to be in place on the relevant buildings on all nights in phase 2 of the experiment. However, in reality there were nights where the installed measures had not been used or were not properly maintained which may, therefore, have allowed badger access. Categorical variables representing individual farms and cameras were incorporated as random effects in the model to account for potential correlation between observations recorded from the same source. Wald tests (using chi-squared statistics) were used to make inference on the main variables and Z-tests were used to make inference on comparisons between different levels of a given variable. Statistical significance was inferred when the associated p-value was less than 5%.

#### Farm level analyses

In order to investigate sources of variation in the likelihood of treatments affecting badger visits to any part of a single farmyard (whether to a specific building or elsewhere), data were aggregated across all cameras for each farm-night. Hence each binary observation in this analysis comprised of a record indicating whether there was photographic evidence of any badgers visiting a given farm on a given night (1) or not (0).

A similar GLMM approach was used to relate variation in the likelihood of a badger visit on any given farm-night to the series of explanatory variables as described above. In order to examine whether there was any displacement of badger activity from protected to unprotected buildings in the farmyard, the effect of treatment status on badger visits was examined at two levels, which were tested independently. First, we tested the effect on badger visits of whether the farm had any exclusion measures in place (regardless of location), compared to where no exclusion measures were in place. Second, the difference in badger visits between the three levels of exclusion treatment (i.e. on feed stores, cattle sheds or both) was investigated. The log_e_ of the number of active cameras was included as a fixed effect covariate as this was analogous to sampling effort and might influence the chance of a positive observation. A term for the individual farm was included as a random effect. All significance-testing was carried out as described above except for post-hoc tests between the different treatments, which were based on chi-squared statistics.

## Results

**Figure 2:**
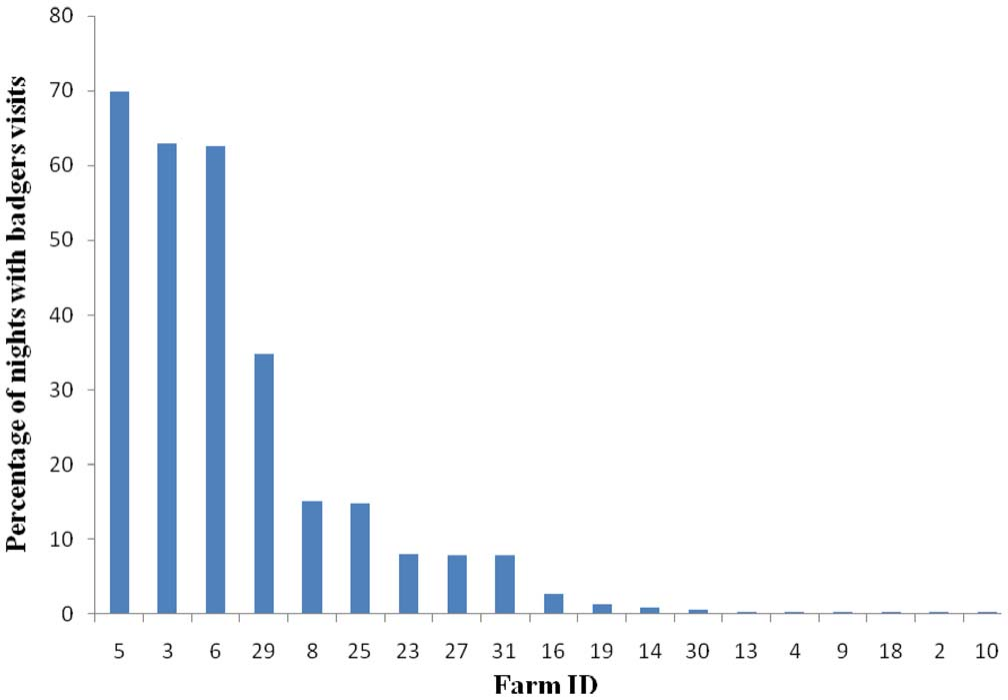
Percentage of nights on which badger visits to farmyards and farm buildings were observed during surveillance phase 1. Observations were made prior to any exclusion measures being installed on study farms.

In phase one (i.e. with no exclusion measures in place on any farms) badger visits occurred on 19 of the 32 farms and on between 0.3% and 71% of the total number of surveillance nights on each farm (Figure 2). Overall, feed storage areas received more than double the number of visits to cattle housing (Table S2). Badger visits to farms occurred throughout the year, but frequency varied significantly with month (GLMM, d.f. = 11, χ^2^ = 142.8, p<0.001). The highest numbers of nights with recorded badger visits were in April, May and June and the lowest in December and January.

The installation of simple exclusion measures on farm buildings significantly reduced levels of badger visits compared to buildings with no protection installed (GLMM, Z = -8.3, p<0.001). Over the two phases, the percentage of nights with incursions into feed stores reduced from 11.2% when no exclusion measures were installed to 0.5% when exclusion measures were installed; for cattle housing the percentage of incursions reduced from 3.5% to 1.2% (Figure S1). With exclusion measures installed there was a highly significant reduction in the frequency of visits to all types of facility, though the reduction in entry to feed stores was greater than in cattle housing (Table 2).

**Table 2:** Results of a GLMM to identify factors associated with variations in the number of nights with badger entry into buildings.

			factors		levels	
Variable	Level	Number of nights with badger visits/Number of nights surveyed (%)	beta	Chi-square (df)	Z-statistic (1 df)	p-value
**Season**				156.4 (3)		<0.001
	spring	546/4048 (13.5%)	0			
	summer	346/4075 (8.5%)	−0.74		−8.6	<0.001
	autumn	240/3458 (6.9%)	−0.96		−10.1	<0.001
	winter	213/3425 (6.2%)	−0.95		−9.8	<0.001
**Phase**	1	738/7111 (10.4%)	0			
	2	607/7895 (7.7%)	+0.51		4.5	<0.001
**Treatment status on night of observation**						
	Treatment vs. No Treatment		−2.02		−8.3	<0.001
	Difference between three treatments			39.8 (2)		<0.001
	**Individual treatment effects**					
	No treatment	1066/9238 (11.54%)	0			
	CH	175/1699 (10.30%)	−1.34		−7.7	<0.001
	FS	70/2421 (2.89%)	−2.62		−13.3	<0.001
	B	34/1648 (2.06%)	−2.02		−8.3	<0.001
	**post-hoc comparisons**					
	FS vs. CH		−1.28	32.4 (1)		<0.001
	FS vs. B		−0.60	7.6 (1)		0.01
	CH vs. B		+0.68	10.5 (1)		0.001

CH  =  Cattle Housing, FS  =  Feed Store, B  =  Both building types, C  =  Control.

During phase two of the experiment there were only 58 recorded entries into buildings which had exclusion measures installed. All of these incursions could be attributed either to the measure not being adequately employed (7 occasions) or maintained (51 occasions). This latter category also included occasions when badger access was possible through damage to other areas of the buildings which had not been repaired. Badger incursions into farm buildings were completely eliminated when exclusion measures were in place and were adequately maintained.

The frequency of badger visits to farms as a whole (both incursions into buildings and observations anywhere in the farmyard) declined significantly when exclusion measures were installed anywhere on a farm (Table 3). Furthermore, the presence of exclusion measures on both feed stores and cattle housing resulted in a significantly greater protective effect, compared to where they were present on only one type of building (Table 3).

**Table 3:** Results of a GLMM to identify factors associated with variations in the number of nights with any badger visits, including both incursions into buildings and observations of badgers within the farmyard (but not entering buildings).

			factors		levels	
variable	level	Number of nights with badger visits/Number of nights surveyed (%)	beta	Chi-square (df)	Z-statistic (1 df)	p-value
**Season**				184.7 (3)		<0.001
	Spring	759/4048 (18.75%)	0			
	Summer	583/4075 (14.31%)	−0.51		−7.0	<0.001
	Autumn	414/3458 (11.97%)	−0.73		−9.1	<0.001
	Winter	299/3425 (8.73%)	−1.09		−12.8	<0.001
**Phase**						
	1	1095/7111 (15.4%)	0			
	2	960/7895 (12.2%)	+0.54		4.9	<0.001
**Treatment status on night of observation**						
	Treatment vs. No Treatment		−2.28		−12.4	<0.001
	Difference between three treatments			31.6 (2)		<0.001
	**Individual treatment effects**					
	No treatment	1465/9238 (15.9%)	0			
	CH	239/1699 (14.17%)	−1.60		−10.0	<0.001
	FS	240/2421 (9.9%)	−1.25		−8.0	<0.001
	B	111/1648 (6.7%)	−2.28		−12.4	<0.001
	**post-hoc comparisons**					
	FS vs. CH		+0.35	3.1 (1)		0.1
	FS vs. B		+1.02	27.6 (1)		<0.001
	CH vs. B		+0.68	12.2 (1)		<0.001

CH  =  Cattle Housing, FS  =  Feed Store, B  =  Both building types, C  =  Control.

The installation of exclusion measures on some buildings also resulted in a significant reduction in recorded incursions into unprotected buildings on the same farm (GLMM, Z  =  −6.1, p<0.001). Incursions into buildings on farms with no measures installed occurred on 2.6% of all nights surveyed whereas incursions into unprotected buildings on farms with measures installed elsewhere on the farm occurred on 2.1% of nights. (Figure S1). While the number of visits to unprotected buildings was significantly reduced by installing measures on either feed stores or cattle housing, the reduction in visits to cattle housing when measures were only installed on feed stores was greater than *vice versa*.

**Figure 3:**
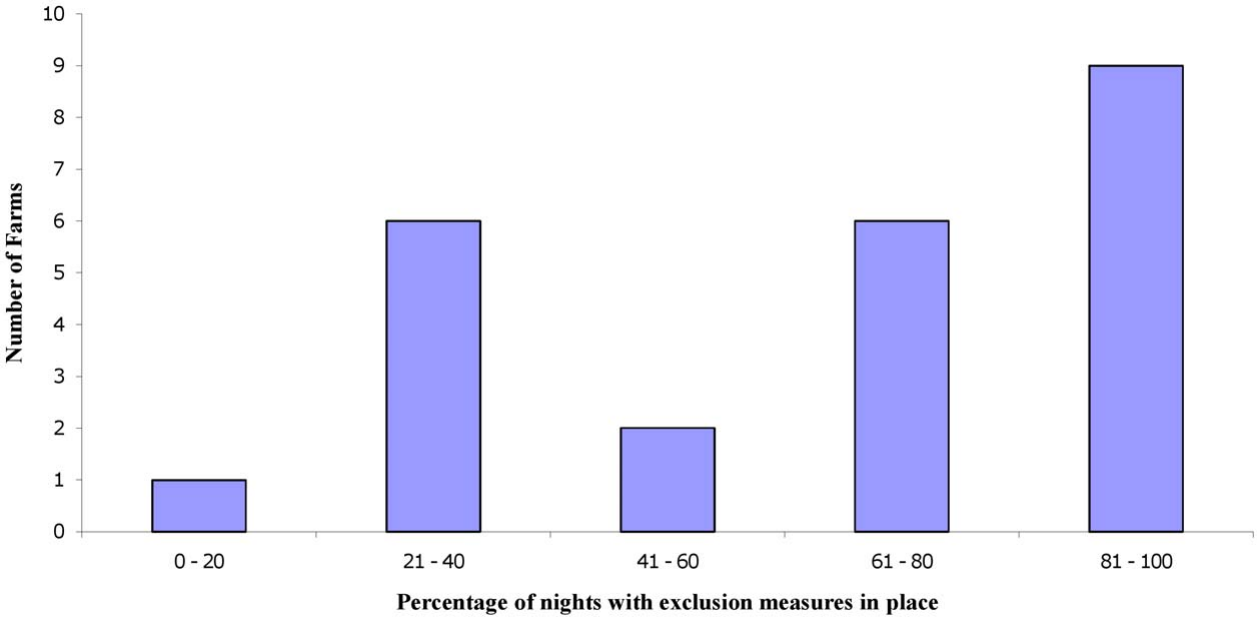
Frequency distribution showing the percentage of surveillance nights on which exclusion measures were observed to be adequately employed. This includes permanent, non-moveable measures, which will always be observed to be in use unless damaged.

The percentage of nights when exclusion measures were adequately employed and maintained varied considerably among farms (from 12% to 98%). However, over half the farms with measures installed (13/24) employed them on over 60% of nights (Figure 3). The results of a simple linear regression indicated that there was no relationship between the frequency of badger visits to a farm in the first phase of the study and the level of farmer compliance during the second (*F*_1,22_  = 2.2, p = 0.2).

## Discussion

This study provides the clearest evidence to date that, in this region, badger visits to farm buildings are a common occurrence. Intensive surveillance over a full year demonstrated that badgers visited buildings at least occasionally on 19 of 32 (59%) farms in our sample. On 3 of the 32 farms (approximately 1 in 10), visits were very frequent, occurring on more than 60% of nights. Badgers visited feed stores and cattle housing, with visits to feed stores being more frequent. While badger visits to farmyards occurred all year round, they peaked in late spring/early summer.

Badgers were successfully excluded from farm buildings with the use of relatively simple, practical exclusion measures. These measures were 100% effective in preventing badger entry into farm buildings when properly used and maintained, such that the only recorded incursions occurred when measures were not employed adequately. Furthermore, the installation of exclusion measures not only stopped entry into buildings but also reduced the level of badger visits to the farmyard as a whole.

The reduction in visits to the farmyard which accompanied protection of one building type (i.e. just feed stores or just cattle housing) was most evident when feed stores were protected. This apparent ‘deterrent effect’, was also observed by Tolhurst et al.[32], who found that the use of electric fencing around feed stores resulted in a reduction in visits to unfenced facilities on the same farms. Tolhurst et al. also radio-tracked the badgers using these farms and demonstrated that excluded badgers simply exploited other food sources within their pre-existing territories, suggesting that farm-derived food may not be vital for the local badger population, at least not in the short term. This hypothesis may be further supported by our finding that installation of exclusion measures reduced the overall level of visits to the farmyard, indicating that when cattle feed is not readily accessible badgers may spend more time in other areas of their territories rather than persistently attempting to gain access to farm-derived feed. If farms were an essential source of food it would be expected that badgers would increase their attempts to gain access to stored feed or, alternatively, that their attentions would turn from protected to unprotected buildings, but neither phenomenon was observed here.

From the camera trap images it was possible to determine that badgers were only able to enter buildings that had exclusion measures installed when the measures were not adequately employed. For example, when gates were left open, when adjustable panels/flaps were not lowered sufficiently or when a new potential entrance point appeared in the building and was not repaired. On average, farmers only used badger exclusion measures that were installed on their farms on approximately 59% of nights, while electric fencing was only used on 48% of nights. On one farm, the retractable electric fencing was only used on 7% of nights. One farmer completely removed some gates that had been installed and on two other farms, walls were almost completely destroyed by cattle or machinery but were not rebuilt, thus negating the exclusion measures that had been installed.

Previous studies have found that farmers rarely employ measures to reduce direct and indirect contact opportunities between badgers and livestock [23], . In the present study exclusion measures were purchased and installed at no cost to the farmer, and yet the extent to which they were adequately employed varied widely, with some farmers diligently using measures almost every night, and others deploying them only rarely. This variation was not related to the background level of badger activity observed during the first phase of our study, even though farmers had been made aware that badgers were visiting their buildings. Measures that required adjustments to existing working practices (e.g. pulling retractable electric fences across, closing feed bin lids, dropping flaps on gates or shutting a gate that was previously not operational) were less likely to be used consistently, as were those that required maintenance (e.g. retractable or fixed electric fencing). Solid metal gates that were installed where gates had previously been situated were used most consistently.

The size and design of farmyards and buildings varies widely, so whilst a suite of badger exclusion measures are available, the number, distribution and nature of their deployment will differ among farms. The uniqueness of each farm also makes it impossible to quote a standard cost for the implementation of badger exclusion measures. For the farms in our study in 2008 the costs of installing exclusion measures ranged from approximately £600 to £12500, with an average cost for their purchase and installation on both cattle housing and feed stores of £3840 per farm. However, this figure should be used with caution as it is derived from a small sample size (n = 8) and costs will vary widely amongst farms depending on their individual characteristics. By comparison, the average cost of a cattle herd breakdown (CHB) in 2010/11 was estimated at £30,000 [35]. Unfortunately, it is not currently possible to conduct a cost-benefit analysis for the installation of badger biosecurity measures as we have no data on the contribution of such measures towards reducing risk of TB in cattle. Due to the relatively small sample size and short duration of the study described here, even if all breakdowns were prevented solely by the use of exclusion measures, there would be insufficient statistical power to detect any significant effect on cattle disease incidence. Nevertheless, intuitively, reducing the potential for direct or indirect contact between badgers and cattle should reduce the risk of disease transmission between the two species.

### Conclusions

Wildlife populations can be a source of infectious diseases of importance to livestock. Where opportunities for transmission arise because of direct or indirect contact in well-defined areas then management of disease risks by using physical barriers may be a practical option. This study clearly demonstrates how relatively simple practical measures can substantially reduce the likelihood of badger visits to buildings. Given the opportunities that visits to farm facilities may present for the transmission of *M. bovis* between badgers and cattle, these measures could potentially have an important role to play in reducing the incidence of TB in cattle. However, we observed wide variation in the extent to which exclusion measures were employed by farmers. In addition, the frequency of badger visits amongst farms varied independently of the presence of exclusion measures, suggesting that badgers are more attracted to some farms than to others and hence that the potential benefits of exclusion measures will also vary. Consequently, the identification of factors that might determine the likelihood of badger visits to farm premises would be a useful aid to individual farmers in making decisions about whether to spend their time and money on installing and maintaining badger exclusion measures.

## Supporting Information

Figure S1 **The percentage of total surveillance nights over both phases when badger incursions into buildings were recorded with (▪) and without (▪) exclusion measures in place.**
(TIF)

Table S1 **Description of exclusion measures installed on each farm.**
(DOCX)

Table S2 **The number of nights when badgers visited the farm (but not necessarily entering farm buildings), entered cattle housing or entered feed stores in both phases.** Values in brackets are percentage of nights surveyed with badger visits.
(DOC)
