# Supplementary figures and images for: Effectiveness of Biosecurity Measures in Preventing Badger Visits to Farm Buildings
Source: PLoS One. 2011 Dec 29;6(12):e28941. doi: 10.1371/journal.pone.0028941 (PMC3248415; doi:10.1371/journal.pone.0028941)

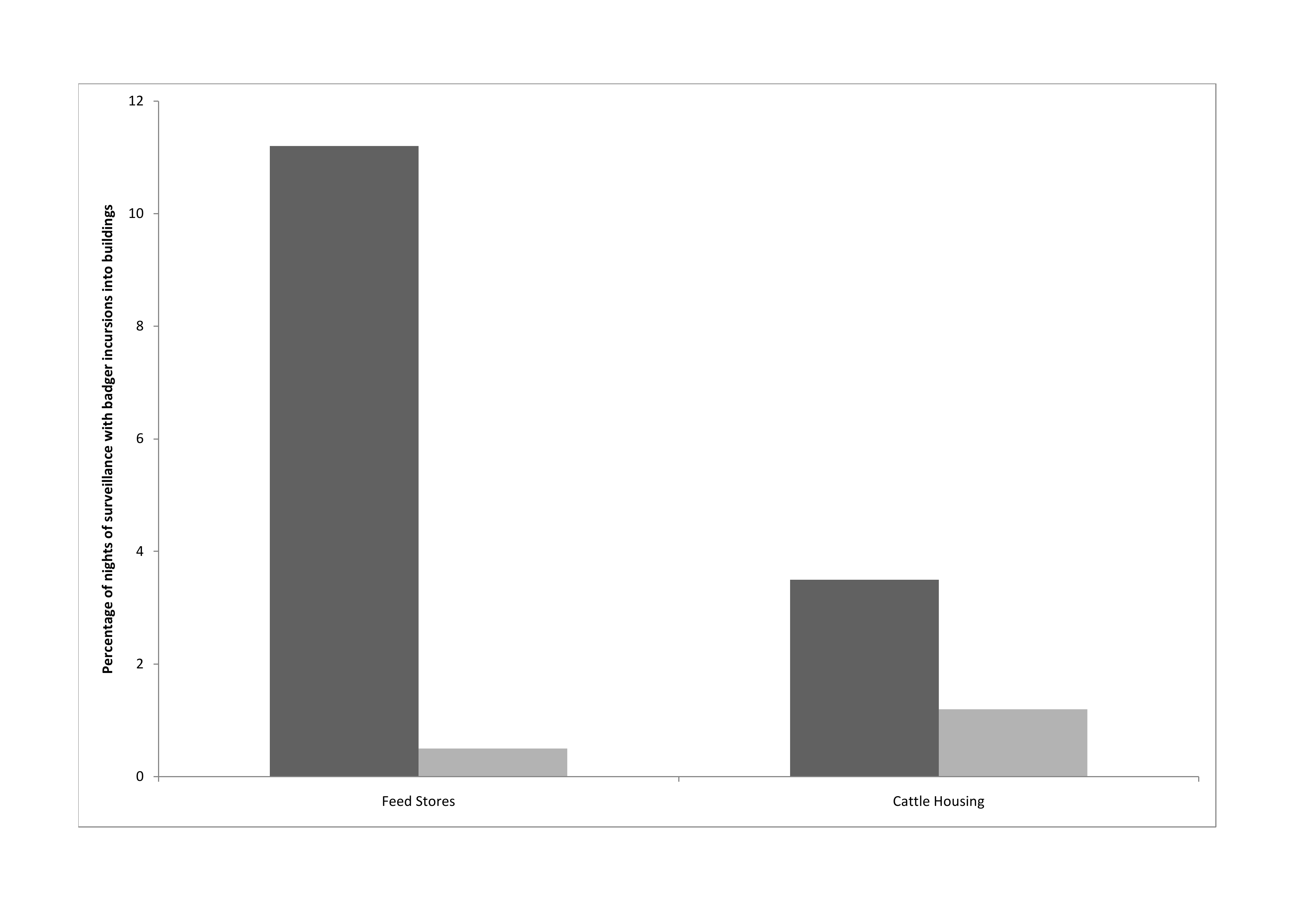

Supplement: Figure S1 — The percentage of total surveillance nights over both phases when badger incursions into buildings were recorded with (▪) and without (▪) exclusion measures in place. (TIF) [file pone.0028941.s001.tif]
