# Supplementary material for: Effectiveness of Biosecurity Measures in Preventing Badger Visits to Farm Buildings
Source: PLoS One. 2011 Dec 29;6(12):e28941. doi: 10.1371/journal.pone.0028941 (PMC3248415; doi:10.1371/journal.pone.0028941)
Supplement: Table S1 — Description of exclusion measures installed on each farm. (DOCX) [file pone.0028941.s002.docx]

**Table S.1. Description of exclusion measures installed on each farm**

Number and type of measure installed varied greatly dependent on size of farm, number of buildings of each type, building construction and farmer preference.

| **Farm ID** | **Treatment** | **Exclusion measures installed** |
| --- | --- | --- |
| 1 | Both | Solid aluminium gates x3  Adaptation of gates *in situ* to reduce gaps |
| 6 | Both | Solid aluminium gates x5  Rail gates with adjustable solid panels x 3  Permanent electric fencing |
| 10 | Both | Adapted solid aluminium wheeled hurdles x 11  Rail gates with solid panels x 2 |
| 20 | Both | Solid and adjustable panels fitted to current gates x10 |
| 26 | Both | Solid aluminium gates x 2  Retractable electric fence x 1  Solid panels fitted to current gates x 4 |
| 27 | Both | Solid aluminium gates x 2  Bespoke solid door to feed store  Solid panelling fitted to wooden rail fence x 6 |
| 31 | Both | Solid aluminium gates x 2  Solid panelling fitted to wooden rail fence x 8 |
| 9 | Both | Rail gates with adjustable solid panels x 4  Adaptation of gates *in situ* to reduce gaps |
| 15 | Cattle Housing | Solid aluminium gates x 17  Solid panelling fitted to wooden rail fence x 6  Retractable electric fence x 1  Rail gates with adjustable solid panels x 4 |
| 5 | Cattle Housing | Solid aluminium gates x 2  Rail gates with adjustable solid panels x 3  Permanent electric fencing x 1  Adaptation of gates *in situ* to reduce gaps |
| 13 | Cattle Housing | Rail gates with adjustable solid panels x 3  Adaptation of gates *in situ* to reduce gaps |
| 17 | Cattle Housing | Solid aluminium gates x 1  Rail gates with adjustable solid panels x 4 |
| 19 | Cattle Housing | Solid aluminium gates x 2  Panelling fitted to hurdles already in place x 4  Permanent electric fence x 1 |
| 21 | Cattle Housing | Solid aluminium gates x 2  Rail gates with adjustable solid panels x 2  Adaptation of gates *in situ* to reduce gaps |
| 23 | Cattle Housing | Solid aluminium gates x 1  Adapted solid aluminium wheeled hurdles x 2  Permanent electric fence x 1  Adaptation of gates *in situ* to reduce gaps |
| 28 | Cattle Housing | Solid aluminium gates x 3  Rail gates with adjustable solid panels x 2  Adaptation of gates *in situ* to reduce gaps |
| 32 | Feed Stores | Solid aluminium gates x 2 |
| 2 | Feed Stores | Adaptation of gates *in situ* to reduce gaps |
| 3 | Feed Stores | Solid aluminium gates x 3  Solid panelling fitted to rail gates x 3  Permanent electric fence x 1  Bespoke solid door to feed store |
| 4 | Feed Stores | Permanent electric fence x 1  Retractable electric fence x 1 |
| 11 | Feed Stores | Solid aluminium gates x 2  Retractable electric fence x 2  Bespoke front & top opening secure metal feed bin |
| 16 | Feed Stores | Feed bin  Adaptation of gates *in situ* to reduce gaps |
| 22 | Feed Stores | Adapted solid aluminium wheeled hurdles x 12 |
| 25 | Feed Stores | Solid aluminium gates x 2  Roller door  Retractable electric fence x 2 |
