# Supplementary material for: Effectiveness of Biosecurity Measures in Preventing Badger Visits to Farm Buildings
Source: PLoS One. 2011 Dec 29;6(12):e28941. doi: 10.1371/journal.pone.0028941 (PMC3248415; doi:10.1371/journal.pone.0028941)
Supplement: Table S2 — The number of nights when badgers visited the farm (but not necessarily entering farm buildings), entered cattle housing or entered feed stores in both phases. Values in brackets are percentage of nights surveyed with badger visits. (DOC) [file pone.0028941.s003.doc]

**Table S2. The number of nights when badgers visited the farm (but not necessarily entering farm buildings), entered cattle housing or entered feed stores in both phases. Values in brackets are percentage of nights surveyed with badger visits.**

|  | **Phase 1** | | | | **Phase 2** | | | | |
| --- | --- | --- | --- | --- | --- | --- | --- | --- | --- |
| **Farm ID** | **No. nights surveyed** | **All Badger Visits (%)** | **Incursions into Cattle Housing (%)** | **Incursions into Feed Stores (%)** | **Treatment Group** | **No. nights surveyed** | **All Badger Visits (%)** | **Incursions into Cattle Housing (%)** | **Incursions into Feed Stores (%)** |
| 1 | 382 | 0 (0) | 0 (0) | 0 (0) | **B** | 368 | 0 (0) | 0 (0) | 0 (0) |
| 6 | 377 | 236 (62.6) | 25 (6.6) | 67 (17.8) | **B** | 429 | 94 (21.9) | 2 (0.5) | 94 (7.5) |
| 9 | 366 | 1 (0.3) | 0 (0) | 1 (0.3) | **B** | 365 | 0 (0) | 0 (0) | 0 (0) |
| 10 | 387 | 1 (0.3) | 0 (0) | 1 (0.3) | **B** | 368 | 3 (0.8) | 0 (0) | 3 (0) |
| 20 | 368 | 0 (0) | 0 (0) | 0 (0) | **B** | 366 | 0 (0) | 0 (0) | 0 (0) |
| 26 | 376 | 0 (0) | 0 (0) | 0 (0) | **B** | 371 | 1 (0.3) | 0 (0) | 1 (0) |
| 27 | 393 | 31 (7.9) | 1 (0.3) | 8 (2) | **B** | 486 | 14 (2.9) | 0 (0) | 14 (0.2) |
| 31 | 384 | 30 (7.8) | 5 (1.3) | 0 (0) | **B** | 365 | 0 (0) | 0 (0) | 0 (0) |
| 7 | 365 | 0 (0) | 0 (0) | 0 (0) | **C** | 370 | 0 (0) | 0 (0) | 0 (0) |
| 8 | 366 | 55 (15) | 33 (9) | 18 (4.9) | **C** | 510 | 183 (35.9) | 148 (29) | 183 (3.1) |
| 12 | 366 | 0 (0) | 0 (0) | 0 (0) | **C** | 365 | 0 (0) | 0 (0) | 0 (0) |
| 14 | 367 | 3 (0.8) | 2 (0.5) | 0 (0) | **C** | 370 | 2 (0.5) | 0 (0) | 2 (0) |
| 18 | 366 | 1 (0.3) | 1 (0.3) | 0 (0) | **C** | 373 | 0 (0) | 0 (0) | 0 (0) |
| 24 | 366 | 0 (0) | 0 (0) | 0 (0) | **C** | 367 | 0 (0) | 0 (0) | 0 (0) |
| 29 | 366 | 127 (34.7) | 113 (30.9) | 16 (4.4) | **C** | 510 | 184 (36.1) | 164 (32.2) | 184 (1) |
| 30 | 366 | 2 (0.5) | 0 (0) | 0 (0) | **C** | 368 | 1 (0.3) | 0 (0) | 1 (0.3) |
| 5 | 366 | 256 (69.9) | 146 (39.9) | 49 (13.4) | **CH** | 477 | 153 (32.1) | 142 (29.8) | 153 (1) |
| 13 | 387 | 1 (0.3) | 1 (0.3) | 0 (0) | **CH** | 367 | 0 (0) | 0 (0) | 0 (0) |
| 15 | 393 | 0 (0) | 0 (0) | 0 (0) | **CH** | 368 | 0 (0) | 0 (0) | 0 (0) |
| 17 | 386 | 0 (0) | 0 (0) | 0 (0) | **CH** | 370 | 0 (0) | 0 (0) | 0 (0) |
| 19 | 396 | 5 (1.3) | 0 (0) | 4 (1) | **CH** | 478 | 83 (17.4) | 27 (5.6) | 83 (0.2) |
| 21 | 369 | 0 (0) | 0 (0) | 0 (0) | **CH** | 390 | 46 (11.8) | 0 (0) | 46 (0) |
| 23 | 365 | 29 (7.9) | 28 (7.7) | 3 (0.8) | **CH** | 379 | 3 (0.8) | 3 (0.8) | 3 (0) |
| 28 | 396 | 0 (0) | 0 (0) | 0 (0) | **CH** | 369 | 0 (0) | 0 (0) | 0 (0) |
| 2 | 370 | 1 (0.3) | 1 (0.3) | 0 (0) | **FS** | 365 | 1 (0.3) | 0 (0) | 1 (0) |
| 3 | 394 | 248 (62.9) | 192 (48.7) | 70 (17.8) | **FS** | 406 | 118 (29.1) | 0 (0) | 118 (9.9) |
| 4 | 366 | 1 (0.3) | 1 (0.3) | 0 (0) | **FS** | 421 | 0 (0) | 0 (0) | 0 (0) |
| 11 | 392 | 0 (0) | 0 (0) | 0 (0) | **FS** | 374 | 0 (0) | 0 (0) | 0 (0) |
| 16 | 377 | 10 (2.7) | 3 (0.8) | 0 (0) | **FS** | 494 | 73 (14.8) | 2 (0.4) | 73 (1.8) |
| 22 | 394 | 0 (0) | 0 (0) | 0 (0) | **FS** | 367 | 0 (0) | 0 (0) | 0 (0) |
| 25 | 388 | 57 (14.7) | 5 (1.3) | 13 (3.4) | **FS** | 371 | 48 (12.9) | 15 (4) | 48 (1.3) |
| 32 | 376 | 0 (0) | 0 (0) | 0 (0) | **FS** | 367 | 0 (0) | 0 (0) | 0 (0) |
